# Chlorophyllides: Preparation, Purification, and Application

**DOI:** 10.3390/biom11081115

**Published:** 2021-07-28

**Authors:** Yi-Ting Wang, Chih-Hui Yang, Keng-Shiang Huang, Jei-Fu Shaw

**Affiliations:** 1 Department of Biological Science and Technology, I-Shou University, Kaohsiung 82445, Taiwan; teengina1220@isu.edu.tw (Y.-T.W.); chyang@isu.edu.tw (C.-H.Y.); 2 Pharmacy Department of E-Da Hospital, Kaohsiung 82445, Taiwan; 3 Taiwan Instrument Research Institute, National Applied Research Laboratories, Taipei 106214, Taiwan; 4 The School of Chinese Medicine for Post-Baccalaureate, I-Shou University, Kaohsiung 82445, Taiwan

**Keywords:** chlorophyllides, chlorophylls, chlorophyllase

## Abstract

Chlorophyllides can be found in photosynthetic organisms. Generally, chlorophyllides have *a*-, *b*-, *c*-, *d*-, and *f*-type derivatives, and all chlorophyllides have a tetrapyrrole structure with a Mg ion at the center and a fifth isocyclic pentanone. Chlorophyllide *a* can be synthesized from protochlorophyllide *a*, divinyl chlorophyllide *a*, or chlorophyll. In addition, chlorophyllide *a* can be transformed into chlorophyllide *b*, chlorophyllide *d*, or chlorophyllide *f*. Chlorophyllide *c* can be synthesized from protochlorophyllide *a* or divinyl protochlorophyllide *a*. Chlorophyllides have been extensively used in food, medicine, and pharmaceutical applications. Furthermore, chlorophyllides exhibit many biological activities, such as anti-growth, antimicrobial, antiviral, antipathogenic, and antiproliferative activity. The photosensitivity of chlorophyllides that is applied in mercury electrodes and sensors were discussed. This article is the first detailed review dedicated specifically to chlorophyllides. Thus, this review aims to describe the definition of chlorophyllides, biosynthetic routes of chlorophyllides, purification of chlorophyllides, and applications of chlorophyllides.

## 1. Definition

Chlorophyllide can be found in photosynthetic organisms. Chlorophyllides are precursors of chlorophylls that play crucial roles in photosynthesis [1,2]. Generally, the concentration of chlorophyllides is low in green leaves [3]. For example, chlorophyllide *a* levels were 182, 92, and 78 nmol/g fresh weight in *Melia azedarach*, *Pisum sativum*, and *Citrus sinensis* leaves, respectively [4]. Chlorophyll *a* levels were 1569, 1064, and 402 nmol/g fresh weight in *Melia azedarach*, *Pisum sativum*, and *Citrus sinensis* leaves, respectively. Chlorophyllides are phytol-free chlorophylls used in the photosynthesis of cyanobacteria [5], green algae [6], and plants [7,8]. The nomenclature of chlorophyllide was first described by Willstatter and Stoll in 1911. They described that the monobasic acid formed through the hydrolysis of chlorophyll was named chlorophyllide [9]. Without the phytyl tail, chlorophyllides are more water-soluble than chlorophyll [10].

Tetrapyrroles are important structure components for hemes, siroheme, chlorophylls, corrins, billins, or coenzymes F_430_ [2,11,12,13]. Chlorophyllides or their derivatives have exhibited diverse applications in agricultural, medical, food colorants, and biotechnological fields [14,15,16,17,18,19]. Generally, chlorophyllides have *a*-, *b*-, *c*-, *d*-, and *f*-type derivatives, and all chlorophyllides have a tetrapyrrole structure with a Mg ion at the center and a fifth isocyclic pentanone. The structure of chlorophyllides is shown in Table 1. Chlorophyllide *a* is generated by the cleavage of the phytol chain from chlorophyll *a* and has a chlorin ring structure with a monomethyl ester at C13^2^. Either ethyl or methyl chlorophyllide *a* is a common ester of chlorophyllide *a* when chlorophyllase acts on chlorophyll in ethanol or methanol [20,21]. 3,8-divinyl chlorophyllide *a* differs from chlorophyllide *a* by having an 8-vinyl group instead of an 8-ethyl group. 3,8-divinyl-chlorophyllide is 8-deethyl-8-vinyl-chlorophyllide, which also can be written as [8-vinyl]-chlorophyllide, with the bracket indicating that a vinyl group has replaced the original substituent at that position. Chlorophyllide *b* can be detected in higher plants, and was first found from greening cucumber cotyledons [22]. Chlorophyllide *b* can be produced from chlorophyllide *a* by chlorophyllide *a* oxygenase (CAO) [23,24]. Chlorophyllide *b* is different from chlorophyllide *a* in a formyl group at C7 [25]. Chlorophyllide *c* is a form of chlorophyllide found in certain marine algae [26,27]. However, the same structure has two different names; some refer to it as “chlorophyll *c*” and some refer to it as “chlorophyllide *c*” [26,27,28]. Generally, chlorophyllide *c* has a porphyrin ring with an acrylate residue, and it can be further divided into chlorophyll(ide) *c*_1_, chlorophyll(ide) *c*_2_ [26,27,29], chlorophyll *c*_3_ [28,30,31], [8-ethyl]-chlorophyll *c*_3_ [32] and *c*_CS-170_ [33]. Compared with chlorophyll(ide) *c*_1_, chlorophyll(ide) *c*_2_ has a vinyl group at C8; chlorophyll *c*_3_ has a vinyl group at C8 and a methoxycarbonyl group at C7. [8-ethyl]-chlorophyll *c*_3_ is different from chlorophyll *c*_3_ with a C8-ethyl group [32]. The C17 of chlorophyll *c*_CS-170_ is a propanoic acid residue [33]. Chlorophyllides *c* existed in a light-harvesting pigment-protein complex where chlorophyllides *c* efficiently transfer absorbed light energy [28,34]. Chlorophyllide *d* is similar to chlorophyllide *a*, except for its C3, which has a formyl group [35,36,37,38]. Regarding chlorophyllide *e*, the nature remains uncertain (e.g., in structure) since it was only described vaguely and was not further characterized [39]. Chlorophyll *f* was first found and named in 2010 by Chen’s group in cyanobacteria [39,40]. The structure of chlorophyllide *f* was predicted using computational studies in 2011 by Yamijala et al. [41]. Generally, chlorophyllide *f* has a chlorin ring structure with a C2-formyl group, and its fluorescence emission is maximal at 722 nanometers (nm).

## 2. Biosynthetic Routes of Chlorophyllides

**Figure 1:**
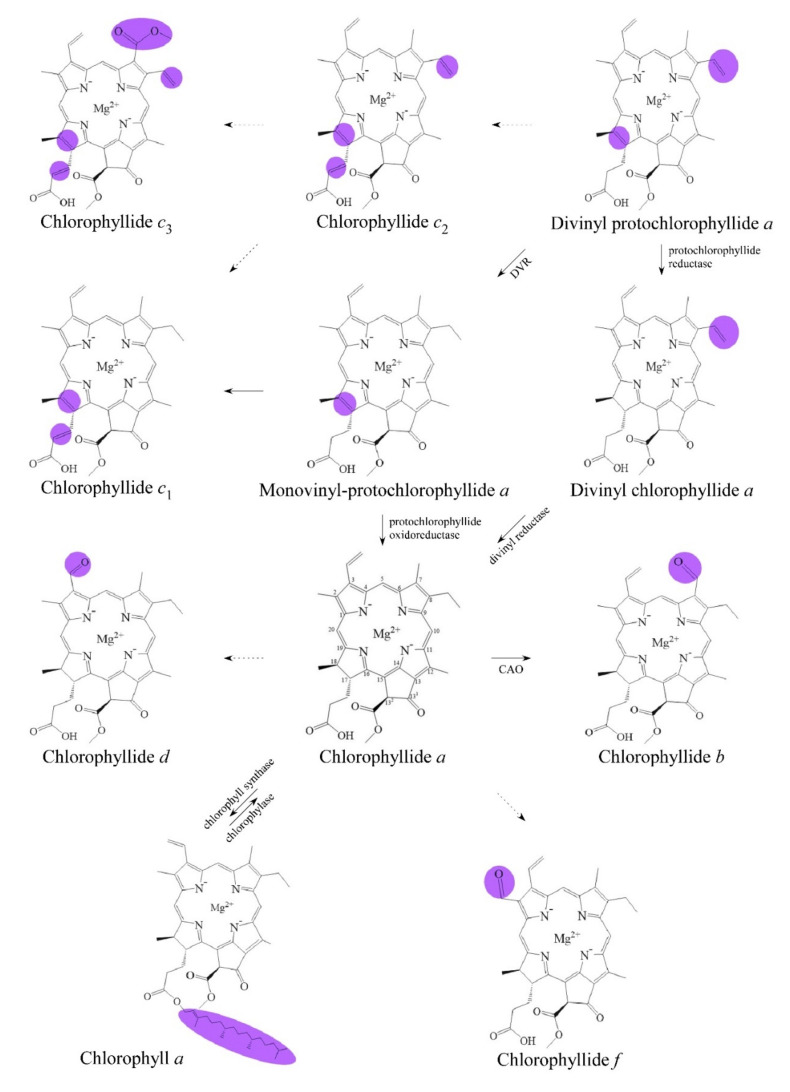
Biosynthetic routes among various chlorophyllides. The structure differences of chlorophyllide derivatives to chlorophyllide *a* are highlighted with purple coloration. 3,8-divinyl protochlorophyllide *a* 8-vinyl reductase: DVR; chlorophyllide *a* oxygenase: CAO.

Details of chlorophyll(ide) biosynthesis have been comprehensively reviewed in several reviews [2,42,43]. The biosynthesis of chlorophyllides in dark-grown plants where protochlorophyllide-to-chlorophyllide phototransformation is the major route of chlorophyll(ide) biosynthesis [44,45]. Here, chlorophyllide is just an intermediate that quickly becomes transformed into chlorophyll and never accumulates in large amounts. Chlorophyllides can be easily produced in large amounts by isolating chlorophyll and then adding chlorophyllase [46,47,48,49,50,51,52,53,54,55,56,57,58]. If the demetallation and removal of phytol are carried out using trifluoroacetic acid or sulfuric acid, pheophorbides are obtained [59]. Although remetalation is much more difficult with pheophorbides, this would be an alternative method for obtaining large amounts of chlorophyllides. The production routes of chlorophyllides are summarized in Figure 1. Generally, chlorophyllide *a* can be obtained from protochlorophyllide *a* (monovinyl protochlorophyllide *a*), divinyl chlorophyllide *a*, or chlorophyll. For example, chlorophyllide *a* can be obtained from protochlorophyllide *a* by protochlorophyllide oxidoreductase [60], 3,8-divinyl chlorophyllide *a* by 3,8-divinyl protochlorophyllide *a* 8-vinyl reductase (DVR) [61], or chlorophyll by chlorophyll chlorophyllidohydrolase (chlorophyllase) [62]. Chlorophyllide *a* can then be transformed into chlorophyllide *b*, chlorophyllide *d*, or chlorophyllide *f*. As for chlorophyllide *c*, it was suggested that divinyl protochlorophyllide *a* or protochlorophyllide *a* could be the precursors owing to their similarities in molecular structure [63,64,65]. It has been reported that divinyl protochlorophyllide *a* and protochlorophyllide *a* could be converted to chlorophyll(ide) *c*_2_ and chlorophyll(ide) *c*_1_, respectively [60,66,67,68]. The biosynthesis pathway of chlorophyllide *d* has not yet been fully elucidated. Chlorophyllide *d* is thought to be derived from chlorophyll *a* or chlorophyllide *a* in genome analysis of *A. marina* [69].

### 2.1. From Protochlorophyllide a to Chlorophyllide a

The biosynthetic pathway of chlorophyllide *a* from protochlorophyllide *a* has been well studied [44,70,71]. It has been reported that protochlorophyllide could be converted to chlorophyllide during light illumination [72,73]. Further studies demonstrated that protochlorophyllide *a* can be accumulated when green leaves are put in the dark. Then, green leaves are exposed to light, and the accumulated protochlorophyllides are then used for the synthesis of chlorophyllide *a* [74]. In 1973, Sundqvist suggested that phototransformation of protochlorophyllide *a* to chlorophyllide *a* depends on the intensity of light [75]. Sundqvist treated wheat leaves with *δ*-aminolevulinic acid for 6 h and then subjected them to light irradiation of 1, 7, 19, 28, and 57 μW/cm^−2^ of light for 15 min. The treatment of *δ*-aminolevulinic acid led to the accumulation of protochlorophyllide_636_ (emission at 636 nm). Protochlorophyllide_636_ was transformed into protochlorophyllide_650_ (emission at 650 nm), and then further photoreduced to chlorophyllide. The obtained chlorophyllides *a* were approximately 2.5, 12.5, 15, 17, and 18 μg/g fresh weight, respectively, suggesting that the intensity of light is correlated to the production of chlorophyllide *a*.

In 1981, Sironval introduced the protochlorophyllide–chlorophyllide cycle [76]. The cycle revealed that protochlorophyllide oxidoreductase catalyzed the protochlorophyllide–chlorophyllide cycle in the presence of nicotinamide adenine dinucleotide phosphate (NADPH). In this cycle, two types of protochlorophyllide oxidoreductase were described: one is dark-operative protochlorophyllide oxidoreductase (DPOR, EC 1.3.7.7), and the other is light-operative protochlorophyllide oxidoreductase (LPOR, EC 1.3.1.33). The catalytic mechanism and structure of protochlorophyllide oxidoreductase were reviewed in several studies [44,77,78,79,80]. The transformation of protochlorophyllide *a* to chlorophyllide *a* was a photochemical reaction that involved two hydrogen atoms added into the tetrapyrrole structure of protochlorophyllide *a*. Schneidewind et al. reported that LPOR is a monomeric apoprotein and formed a ternary complex with protochlorophyllide and NADPH [81]. The assembly of the ternary complex LPOR/NADPH/protochlorophyllide further induced the dimerization of ternary complex. Through the absorption of light by the bound-protochlorophyllide molecule, activated LPOR was responsible for transferring a hydride from the nicotinamide ring of NADPH to the C17 of protochlorophyllide. Sequentially, a proton donated from the tyrosine residue at the active site of LPOR was transferred to the C18 of protochlorophyllide [82,83]. DPOR is a nitrogenase-like enzyme that contained L, B, and N subunits. DPOR contained two separable components: L-protein as the ATP-dependent catalytic component and NB-protein as the reduction of protochlorophyllide [84,85,86]. DPOR transferred a single electron from NB-protein to protochlorophyllide, which produced protochlorophyllide anion radicals. Followed by a single proton transfer, sequential proton- and electron-transfer steps transformed the anion radicals into chlorophyllide. Moreover, Nguyen et al. reported a new mechanism that was coordinated by LPOR between photosynthetic membrane biogenesis and chlorophyll synthesis in plants. Protochlorophyllide and parts of LPOR were inserted into the outer membrane leaflet, which targeted chlorophyllide to the synthetic site of chlorophyll [87].

Spectral forms and photoactivity of protochlorophyllides or chlorophyllides have been studied in the 1950s and in detail later by Sundqvist’s group around the 1990s [88,89,90,91]. In 2000, Schoefs clarified the correlation between photoactive and photoinactive protochlorophyllides, chlorophyllides, NADPH, and light illumination. The difference between photoactive (e.g., P638–645 and P650–657) and photoinactive (e.g., P628–633 and P642–649) protochlorophyllides was their spectral properties. Results indicated that when photoinactive protochlorophyllide combined with NADPH, photoactive protochlorophyllide could be generated. After illumination, photoactive protochlorophyllide could be converted into chlorophyllide [92].

Besides light irradiation, several factors have been studied for chlorophyllide production. For example, Yahubyan et al. reported an effective method for chlorophyllide *a* generation. They used norflurazon, an inhibitor of carotenoid biosynthesis, to diminish the synthesis of carotenoids. Their results showed that more chlorophyllide *a* could be obtained in the presence of norflurazon. The increased amount of chlorophyllide *a* was suggested to be the result of an inhibition of carotenoid biosynthesis, which led to more protochlorophyllide *a* for the synthesis of chlorophyllide *a* [93]. Previous studies have demonstrated that norflurazon globally down-regulated the genes involved in tetrapyrrole biosynthesis [94,95,96]. In addition, water is a key factor. Le Lay et al. studied the effects of dehydration during the transformation of chlorophyllide in etiolated barley leaves (*Hordeum vulgare*) [97]. They observed that dehydration had no effect on the phototransformation of protochlorophyllide into chlorophyllide. In 40% and 78% desiccated leaves, photoactive protochlorophyllide achieved 80% and 28% of the control level, respectively. These results emphasized that dehydration induces a decrease in protochlorophyllide synthesis and ultimately leads to a decrease in chlorophyllide *a* generation. Therefore, the authors identify and distinguish different steps at the dehydration-affected transformation pathway of chlorophyllides. Moreover, the influence of nitric oxide was proposed. Zhang et al. demonstrated that nitric oxide inhibits the formation of chlorophyllide. Barley (*Hordeum vulgare* L. Zaoshu No. 3) seeds were cultured in the dark and treated with nitric oxide. The results demonstrated that nitric oxide treatment decreased the activity of NADPH:protochlorophyllide oxidoreductase, which led to the accumulation of protochlorophyllide and then decreased the formation of chlorophyllide in the barley leaf apex [98].

In Table 2, some factors affecting the formation of chlorophyllide *a* are summarized. Results reveal that light illumination, light intensity, *δ*-aminolevulinic acid, protochlorophyllide oxidoreductase, light-independent protochlorophyllide reductase, norflurazon, and water have positive effects on the production of chlorophyllide, whereas nitric oxide has a negative effect.

### 2.2. From Divinyl-Chlorophyllide a to Chlorophyllide a

Divinyl-chlorophyllide *a* can be converted to chlorophyllide *a* by a DVR enzyme. The DVR enzyme, a divinyl chlorophyllide a-specific and NADPH-dependent enzyme, can convert the C8-vinyl group of divinyl-chlorophyllide *a* to a C8-ethyl group [104]. Various 8-vinyl reductase genes have been identified and characterized in *Arabidopsis thaliana* (*AT5G18660*) [23,105], *Chlorobium tepidum* (*bciA*) [106], *Synechocystis* sp. PCC 6803 (*slr1923*; *bciB*) [107,108], *Oryza sativa* (*Os03g22780*; *OsDVR*) [109], *Cucumis sativus (CsDVR)* [110], *Zea mays* (ZmDVR) [110], *Acaryochloris marina* [111], and *Rhodobacter capsulatus (bciA)* [112].

The DVR enzyme is encoded by the *AT5G18660* gene in *Arabidopsis thaliana*. The *AT5G18660* gene has an open reading frame of 1251 base pairs (bp) and encodes 417 amino acids. The enzyme has 49 amino acids of chloroplast transit peptide that function in chloroplasts. A transmembrane *α*-helix located in the enzyme is located on the membranes. In 2005, the *AT5G18660* gene of *Arabidopsis thaliana* was identified by Nagata et al. [105]. The same year, Nakanishi et al. found that the *AT5G18660* gene mutant exhibited pale-green phenotypes and reduced chlorophyll *b* [113].

In 2007, Chew and Bryant identified the NADPH-dependent 3,8-divinyl protochlorophyllide *a* 8-vinyl-reductase BciA (*bciA*) gene of the green sulfur bacterium *Chlorobium tepidum*, which is homologous to *AT5G18660*, with 75% similarity. Results demonstrated that the recombinant BciA reduced the C8-vinyl group in divinyl protochlorophyllide in the presence of NADPH as the coenzyme [106]. Furthermore, Azai et al. demonstrated that recombinant BciA could reduce the C8-vinyl reduction activity in *Rhodobacter capsulatus* [112].

In 2008, an *slr1923* gene participating in the reduction of the C8-vinyl group in *Synechocystis* sp. PCC 6803 was isolated. Ito et al. demonstrated that the growth rate of the *slr1923* mutant was significantly retarded under high light conditions (150 μmol photons m−2 s−1). Their results showed that the *slr1923* mutant accumulating 3,8-divinyl chlorophyll was sensitive to high light irradiation [108]. In addition, Islam et al. suggested that the knockout *slr1923* mutant lost its ability to synthesize monovinyl chlorophyll(ide) and then accumulated 3,8-divinyl chlorophyll(ide) [107]. Liu and Bryant reported that an *slr1923* ortholog from *Chloroherpeton thalpotassium* could complement the *Chlorobium tepidum* bciA mutant and restore the synthesis of monovinyl chlorophyllide [114]. Therefore, Bryant et al. termed *slr1923* and its homologs as *bciB* [115]. Canniffe et al. heterologously expressed the *bciA* or *bciB* gene in *Rhodobacter sphaeroides* and *Synechocystis* sp. PCC 6803. Their results demonstrated that BciA could reduce divinyl protochlorophyllide and divinyl chlorophyllide, and preferred the reduction of the 8-vinyl group of divinyl-chlorophyllide to others [116].

Previous studies demonstrated that the activity of DVR requires some cofactors as reductants. For example, Parham et al. indicated that DVR (BciA) and NADPH are involved in the reduction of divinyl chlorophyllide *a* to chlorophyllide *a* in cucumber plastids [104]. Whyte and Griffiths further reported that the ratio of NADPH/NADP+ was significantly decreased after the synthesis of chlorophyllide in wheat (*Triticum aestivum var. Avalon*) and barley (*Hordeum vulgare L. var. Proctor*) [61]. In vitro assays performed with DVR (BciA) from various species also showed that NADPH was a reductant for the DVR enzyme [106,109]. Unlike BciA, DVR (BciB) in *Chloroherpeton thalassium* required ferredoxin as an electron donor, NADPH, and ferredoxin–NADP+ oxidoreductase (FNR) [117].

A DVR gene (*Os03g22780*, *OsDVR*) was first identified in rice (*Oryza sativa*) by Wang et al. [109]. The open reading frame of *OsDVR* has 1218 bp and encodes a 405-amino acid protein with a molecular weight of 43 kilodaltons (kDa). The *N*-terminus of *OsDVR* contained a chloroplast-targeting sequence of 58 amino acids. *OsDVR* was found in a mutant *824ys* and encoded a DVR that has 66% similarity to *AT5G18660* in *Arabidopsis*. *OsDVR* of the *824ys* mutant has a deletion of three amino acids located in the *α*-helix transmembrane domain. The *824ys* mutant presented a yellow–green leaf, accumulation of divinyl chlorophyllide, suppressed development of chloroplast thylakoids, and retardation of growth rate. Deng’s group demonstrated that OsDVR could convert divinyl chlorophyllide *a* or divinyl chlorophyll *a* into monovinyl chlorophyllide *a* or monovinyl chlorophyll *a* [109]. They performed further studies on the substrate specificity of DVR from *Arabidopsis thaliana* (AtDVR), *Oryza sativa* (OsDVR), *Cucumis sativus* (CsDVR), and *Zea mays* (ZmDVR). The open reading frame of *CsDVR* contained 1260 bp and encoded a 419-amino acid protein with 46 kDa. The open reading frame of ZmDVR contained 1206 bp and encoded a 401-amino acid protein with 43 kDa. The *N*-terminus of the chloroplast-targeting sequence contained 54 and 71 amino acids. The similarity of *CsDVR* or *ZmDVR* was 62% or 71%, respectively, compared to *AT5G18660*. For divinyl chlorophyllide a, OsDVR and ZmDVR had higher DVR activities than CsDVR and AtDVR [110].

### 2.3. From Chlorophyll to Chlorophyllide a by Chlorophyllase

Many enzymes are involved in the synthesis/degradation pathways of chlorophyllides, such as chlorophyll dephytylases, pheophytinases, chlorophyll synthases, and chlorophyllase [2,11,15]. For example, chlorophyllase is responsible for the hydrolysis of chlorophylls into chlorophyllides [52,118]. Pheophytinase is responsible for the removing the phytol group of pheophytin (Mg-free chlorophyll). Various chlorophyllases have been summarized in the chlorophyllase section of Table 2. Chlorophyllase (EC 3.1.1.14), an esterase found in leaves, was first named by Willstätter and Stoll according to its specific-substrate chlorophyll [9,20]. Chlorophyllase performed hydrolytic and esterification activities [119,120,121]. Chlorophyllase has been known to hydrolyze phytol from chlorophylls to generate chlorophyllides [63]. The final step in the biosynthesis of chlorophyll *a* was esterification with phytol of the propionic acid residue at C7 of chlorophyllide *a*. Chlorophyllase also performed transesterification of chlorophyllides depending on the reaction condition [122,123]. In vitro, a transesterification of methyl-chlorophyllide with geranylgeraniol occurred in the presence of chlorophyllase from *Chlorella protothecoides* [124]. Therefore, in vitro activity of chlorophyllase acted as hydrolase, esterase, and transesterase [51,125,126,127]. Early works also assumed its hydrolytic function in vivo [128]. The quantitative studies of chlorophyllase activity on chlorophyll hydrolysis were first performed by Mayer in 1930 [129]. Generally, the production of chlorophyllide relies on endogenous chlorophyllase [46,47,48,49,50,51,52]. For example, Hsu et al. described an efficient and long-used way to get chlorophyllides. Chlorophylls could be prepared from spinach and subsequent hydrolysis using chlorophyllase from the *Ficus macrocarpa* leaf [99,100].

In 1999, the first chlorophyllase gene was cloned by Jacob-Wilk et al. and Tsuchiya et al. [53,54]. Jacob-Wilk et al. isolated and characterized chlorophyllase from *Citrus sinensis cv. Valencia*. The encoded amino acid sequence of chlorophyllase 1 has 329 residues with a domain of serine-lipases and a putative chloroplast-targeted transit peptide [53]. In the same year, Tsuchiya et al. cloned chlorophyllase (e.g., CaCLH) from *Chenopodium album* and *Arabidopsis thaliana* (e.g., AtCLH1 and AtCLH2). The amino acid identity of amino acid sequences between *Chenopodium album* and *Arabidopsis thaliana* was 32–40%. The highly conserved regions of CaCLH, AtCLH1, and AtCLH2 were the lipase motif and ATP/GTP-binding site motif A. A putative endoplasmic reticulum-targeted signal sequence was found in CaCLH, and a typical chloroplast-targeted signal sequence was found in AtCLH2 [54].

Our group has cloned chlorophyllases from *Brassica oleracea* (e.g., BoCLH1, BoCLH2, and BoCLH3), *Chlamydomonas reinhardtii* (e.g., CrCLH1), and cyanobacterium *Cyanothece* sp. ATCC 51142 (e.g., CyanoCLH) [55,56,57,58]. The full-length cDNAs of BoCLH1, BoCLH2, and BoCLH3 were 1140 bp, 1104 bp, and 884 bp, which encoded 34.7, 35.3, and 23.5 kDa of putative chlorophyllases, respectively [55,56]. Three chlorophyllases (BoCLH1, BoCLH2, BoCLH3) contained a highly conserved lipase motif (GXSXG), but the His residue of the catalytic triad (Ser–His–Asp) was missing in BoCLH3. The *N*-termini of BoCLH1 and BoCLH2 were predicted as chloroplast-targeted signal peptides, whereas BoCLH3 has a plasma membrane-targeted sequence. Kinetic analysis showed that BoCLH1 preferentially hydrolyzed Mg-free chlorophyll (e.g., pheophytin), while BoCLH2 hydrolyzed both chlorophyll and Mg-free chlorophyll (e.g., pheophytin). The cDNA length of CrCLH1 was 969 bp, and the protein molecular mass was 36.5 kDa [57]. CrCLH1 contains a conserved GHSRG lipase motif and a Ser–Asp–His catalytic triad that belongs to the *α*/*β* hydrolase superfamily. For CrCLH1, we demonstrated that the subcellular localization of CrCLH1 was outside of the chloroplast. Kinetic data revealed that CrCLH1 preferred chlorophyll *a* as a substrate to chlorophyll *b* and bacteriochlorophyll *a*. In our previous study, a novel application of chlorophyllase cloned from eubacteria *Cyanothece* sp. ATCC 51142 was used to hydrolyze bacteriochlorophyll [58]. Traditionally, bacteriochlorophyll *a* was treated with trifluoroacetic acid and subsequently subjected to a series of purification processes. This process is time- and labor-consuming and environmentally unfriendly. We used this novel CyanoCLH chlorophyllase to hydrolyze bacteriochlorophyll *a* from *R. sphaeroides*, chlorophyll *a* from *Anacystis nidulans* algae, and chlorophyll *b* from spinach. The results indicated that recombinant CyanoCLH preferred hydrolyzing bacteriochlorophyll *a* from *R. sphaeroides.* The characterization of a chlorophyllase (OaCLH) from the cyanobacterium *Oscillatoria acuminata* PCC 6304 was reported [130]. This study demonstrated that the substrates of recombinant OaCLH were chlorophyll *a*, chlorophyll *b*, bacteriochlorophyll *a*, and pheophytin *a*, and chlorophyll *b* and chlorophyll *a* were especially preferred. Recently, a putative esterase, Slr1916, was found in the mutant of the cyanobacterium *Synechocystis* sp. PCC 6803. This study showed that Slr1916 functioned as chlorophyllase activity in vitro [103]. Therefore, recombinant chlorophyllase could be used as a catalytic reactor for producing bacteriochlorophyllides or chlorophyllides which provide an economic and environmentally friendly method.

The activity of chlorophyllases has been reported in many studies. The level of chlorophyllases is affected by many factors, including detergent, antibiotics, lead, ethylene, and others. For example, McFeeters et al. demonstrated that the activity of chlorophyllase from *Ailanthus altissima* was inhibited by the detergent Triton X-100. Hence, the concentration of Triton X-100 should be kept at 0.2% during the preparation of chlorophyllase from plants [51]. Moll et al. found that after light treatment for 60 min in *Phaseolus vulgaris* L., chlorophyllase activity in leaf extracts was doubled within 3 days, resulting in the synthesis of chlorophyll *a* and *b*. Interestingly, when the plants were exposed to periodic illumination (1 min light–59 min dark), the activity of chlorophyllase was doubled within 1 to 2 days; however, this led to the synthesis of chlorophyll *a* and inhibited the synthesis of chlorophyll *b*. When plants were treated with periodic and continuous light, chlorophyllase activity was decreased and chlorophyll *b* was synthesized. Moll et al. also found that different effects of antibiotics (chloramphenicol) inhibited chlorophyllase activity, while cycloheximide effectively inhibited chlorophyllase activity in light [131].

It has been reported that lead could inhibit the activity of chlorophyllase, leading to increased degradation of chlorophyll [132]. Jacob-Wilk et al. reported that the transcriptional level of chlorophyllase was low during fruit development; however, the level increased with ethylene treatment at all stages of development. The senescence-delaying regulator gibberellin-A3 (GA3) affected the ethylene-induced level of chlorophyllase [53]. Tsuchiya et al. reported that the mRNA of *AtCLH1* was increased and maintained 9 h after treatment with methyl jasmonate in *Arabidopsis* [54]. Results showed that methyl jasmonate increased senescence and the degradation of chlorophyll. Treatment of *δ*-aminolevulinic acid in French bean leaf increased the activity of chlorophyllase in a dose-dependent manner [101]. The post-translational regulation reported by Harpaz-Saad et al. showed that the *N*-terminal 21 amino acid fragment is important to the activity of chlorophyllase [102]. Full-length and truncated chlorophyllase cloned from *Citrus sinensis* was overexpressed in *Nicotiana tabacum*. Results also indicated that plants with the truncated form of chlorophyllase accumulated chlorophyllide, leading to a chlorosis phenotype [102].

## 3. Purification of Chlorophyllides

Generally, chlorophyllides are unstable due to the loss of the central magnesium ion, epimerization, or allomerization. The amphiphilic character of chlorophyllides can be used to distinguish chlorophyllides from chlorophyll and other metabolites. Since chlorophyllide *a* and *b* are the major and abundant forms in plants, many investigations have been focused on chlorophyllide *a* and *b* to date. Herein, we provided a detailed overview about their purifications and applications.

### 3.1. Purification Using Solvents

Previous researchers have used methanol, acetone, *N*,*N*’-dimethylformamide, diethyl ether/ethanol, or *n*-hexane to extract chlorophyllides. Chlorophyllide purification using solvents is shown in Table 3. For example, Peschek et al. isolated plasma membranes and thylakoid membranes of *Anucystis nidulans* [133]. Membrane suspensions were extracted with acetone (or methanol) and 0.1 M ammonium hydroxide (9:1, *v*/*v*)*,* and then petroleum ether or *n*-hexane was added to separate the two phases. Results demonstrated that chlorophyllide *a* existed in aqueous plasma membrane suspension with emission peaks at 674 and 682 nm. Muller et al. extracted chlorophyllide with acetone, *n*-hexane, and diethyl ether [134]. Barley (*H. vulgare* L.) leaves were ground in liquid nitrogen and then suspended in 80% acetone (acetone/50 mM Tricine–NaOH, pH 8, 80:20, *v*/*v*). The suspension was repeatedly extracted with *n*-hexane, added to diethyl ether/ethanol (1:1), and then repeatedly washed with 100 mL of 20% ethanol (10 mM Tricine–NaOH, pH 8). The obtained pigments were chlorophyllides: 17 μg of chlorophyllide *a* and 5 μg of chlorophyllide *b* per gram of fresh leaves.

### 3.2. Purification Using Chromatography

Besides the solvent method, chlorophyllides could be separated using chromatography as shown in Table 4. The separation of chlorophyllide and chlorophyllide derivatives was performed using column chromatography with sugar or cellulose powder [48]. Araki et al. improved on column chromatography to completely separate chlorophyllide and pheophorbide. Crude pigments were prepared from *Porphyra yezoensi* and then chromatographed on a column packed with the acetate form of DEAE-Sepharose CL-6B. The results showed that chlorophyllide could be eluted with acetone/H_2_O (4:1, *v*/*v*) and 1.0% ammonium acetate at 30 min [135]. In addition, Hanamoto and Castelfranco used a silicic acid column coated with dodecyl residues on high-performance liquid chromatography (HPLC) to separate chlorophyllide, divinyl-chlorophyllide, and monovinyl-, and divinyl-protochlorophyllide from cucumber cotyledons at 0 °C. The moving phase was a lipophilic cation and tetrabutyl ammonium. Ion-pair elution was performed using 70% methanol with different concentrations of methyl ethyl ketone. The results indicated that chlorophyllide could be separated at 70 min using this system [136]. Mantoura and Llewellyn developed a reverse-phase HPLC system for rapid separation (~20 min) of chlorophylls, breakdown products, and carotenoids from algal cultures and natural waters. The ion-pairing reagent contained tetrabutylammonium acetate and ammonium acetate that provide good resolution with acidic pigments, such as chlorophyllides and phaeophorbides. The detected limitation of chlorophyllide from this protocol was 0.01–0.2 ng. This method provided good separation of acidic pigments; however, the separation of phaeophorbide *b*, phaeophorbide, and chlorophyllide *b* methyl esters was not reported [137].

Shioi and Beale carried out an HPLC analysis using a polyethylene column [138]. Polyethylene provides excellent separation of mono- and divinyl chlorophyllides and other pigment mixtures without adsorption of the pigment with free carboxylic acid groups. They suggested that the resolution and retention time of separated efficiencies were improved in a polarity-dependent manner (mobile phase). The proportion of optimal acetone in water to separate chlorophyllides was 50% (*v*/*v*) at a flow rate of 0.2 mL/min at 20 °C. This method performed a methanol–water eluent, and acetic acid acted as the ion-pairing agent. This method achieved a better separation; however, chlorophyllides were not completely resolved and the peaks were broad. Zapata et al. applied a reversed-phase column (a prepacked 5 μm Spherisorb ODS-2 column) with HPLC to separate chlorophyllides, chlorophylls, and other degradation products from *Phaeodactylum tricornutum* and *Dunaliella tertiolecta*; the mobile phases contained 80% methanol in ammonium acetate solution and 80% methanol in acetone [139]. Results suggested that ammonium acetate was a better reagent than the ion-pairing reagent owing to its complete resolution of a complex porphyrin mixture. Therefore, eluents containing ammonium acetate were suitable for the reversed-phase separation of more than 20 pigments, including chlorophyllide *a*, which was distinguished at 5 min.

Schoefs et al. separated pigments from bean leaves by reversed-phase HPLC with a photodiode-array detector [140]. The photodiode-array allowed the simultaneous recorded and determined chromatograms at a different wavelength. The bound phase of the reversed column was inert, which prevents the decomposition or structural modification of chlorophyllides. Therefore, the authors identified over twenty plant pigments including protochlorophyllides, chlorophyllides, and carotenoid isomers. Darko et al. further improved the method of reversed-phase liquid chromatography (LC) to separate the pigments from green leaves [141].

Garrido et al. separated chlorophylls and chlorophyllides using HPLC with a monolithic C_18_-bonded silica rod column [142]. The mobile phases contained 80% methanol in 0.025 M aqueous pyridine solution (pH 5 with acetic acid) and 80% methanol in acetone. Monolithic columns contained interconnected small-sized skeletons and pass-through pores, which provided high-speed separation, decreased flow resistance, and reduced diffusion path length. Pyridine has advantages; for example, it formed a homogeneous mixture with water and most organic solvents, it did not interfere with pigment detection, and it did not react with acetone. This method had high flow rates, and chlorophyll *a* and chlorophyllide *a* could be separated in less than 4 min. Kruk and Myśliwa-Kurdziel developed a new HPLC method with a commercial C30 reverse-phase column and isocratic elution [143]. The authors purified monovinyl protochlorophyllides and divinyl protochlorophyllides from submilimolar concentrations of crude protochlorophyllide extract. Although this study did not test the purification of chlorophyllides, the obtained protochlorophyllides could be applied as the material to synthesize chlorophyllides. Loh et al. identified chlorophyll and its derivatives from *Taraxacum formosanum* using HPLC-diode array detection−mass spectrometry with a HyPURITY C18 column [144]. The solvent system consisted of water, methanol, acetonitrile, and acetone with a gradient condition of 1 mL/min flow rate. The authors performed two extraction systems to collect chlorophyllide and other derivatives. Firstly, chlorophyllides and other derivatives were extracted with hexane/ethanol/acetone/toluene (10:6:7:7, *v*/*v*/*v*/*v*). HPLC analysis indicated that 11 chlorophyll derivatives were identified using this extraction protocol. Furthermore, this solvent system was prone to separate dephytylated chlorophyll derivatives, such as chlorophyllide *a* and chlorophyllide *b*. Chlorophyllide *a* was separated at 6.15 min and the content of chlorophyllide *a* was 0.17 μg/g. In the second extraction method, the authors performed a column chromatography containing magnesium oxide-diatomaceous earth (1:3, *w*/*w*) in acetone and 50% ethanol to extract chlorophylls and their derivatives. The analysis indicated that 10 chlorophyll derivatives were identified by this extraction protocol, in which the content of chlorophyllide *a* was 140.92 μg/g.

Chen et al. developed a high-throughput method that combined HPLC and time-of-flight mass spectrometry to distinguish chlorophyllides and their derivatives [145]. Ammonium acetate in water (1 M) was used as the ion reagent, a C18 Spherisorb ODS-2 column was used, and the flow rate was set at 1.0 mL/min in chromatographic separation. The mobile phases were water/ion pair reagent/methanol (1:1:8, *v*/*v*/*v*) and methanol/acetone (1:1, *v*/*v*). Mass spectrometry was performed using atmospheric-pressure chemical ionization or matrix-assisted laser desorption/ionization time-of-flight mass spectrometry. The data obtained from high resolution time-of-flight mass spectrometry determined the accurate mass of chlorophyllide derivatives. The result indicated that 16 of the chlorophyllide derivatives were identified, and chlorophyllide was separated at 7.9 min.

During the purification process of chlorophyll or chlorophyllides, it is important to maintain the central magnesium ion and prevent the disruption of the tetrapyrrole macrocycle. The position of C13^2^ in chlorophyll led to the relatively high acidity and high reactivity. At the chiral C13^2^-position, chlorophyll(ide)s are transformed to epimers via stereoinversion and allomers via oxidation [146,147,148]. It is well known that the epimerization and allomerization occur easily under basic conditions and an alcoholic solution [149,150]. Hynninen et al. reported a free-radical allomerization mechanism of chlorophyll [151]. Hynninen and Hyvärinen further demonstrated the allomerization pathways of chlorophylls under essentially anhydrous chlorophyll and thoroughly dried methanol [150]. The treatment of 2–5% NaOH from 7 to 4 h increased the formation of allomerized derivatives of chlorophyll [149,152,153]. Some examples of chlorophyllides purification by chromatography are shown in Table 4.

## 4. Applications of Chlorophyllides

### 4.1. Various Biological Activities

The bioavailability of chlorophyllides and their derivatives has been well discussed. For example, Hayes and Ferruzzi exhibited that the bioavailability of chlorophyllide derivatives was dependent on the polarity and reactivity of the molecules [154]. In addition, the uptake (intestinal, etc.), biotoxicity, and the critical assessment of in vitro assays and in vivo effects have also been studied. For example, Szczygieł et al. investigated uptake, distribution, and clearance of chlorophyllide *a* and Zn-chlorophyllide *a* in tumor-bearing mice. The results showed that uptake and clearance of chlorophyllide *a* were faster than Zn-chlorophyllide [18]. The higher content of chlorophyllide *a* was detected after 0.5 h of administration. Chlorophyllide *a* showed tissue selectivity as a higher affinity in the liver and intestine. Hsu et al. studied the organ-specific distribution of chlorophyllide in rabbits and revealed that the peak values of chlorophyllide *a* in the gallbladder and liver were 2 h and 8 h post feeding, respectively [155]. Additionally, chlorophyllides were prone to absorb over chlorophyll in Caco-2 cell studies [156,157]. Zhong et al. also reviewed the bioactivity and mechanisms of chlorophyll and metallo-chlorophyll derivatives (copper-, zinc- and iron-chlorophyll) during absorption, distribution, metabolism, and excretion in vitro and in vivo [158].

Chlorophyllides exhibit many biological activities, such as anti-growth, antimicrobial, antiviral, antipathogenic, and antiproliferative activity. For example, in 1954, Blaauw-Jansen explained that chlorophyllide could be used as a potential precursor of growth inhibitors [159]. The author spotted an ether extract of *Chlorella* on a paper strip to perform paper chromatography. The paper strips were then used to test its inhibition zone against *Staphylococcus aureus*, the spots of chlorophyllides with *R_F_* = 0 showing on the inhibition zone. After exposure to light, chlorophyllides were bleached, and the inhibition became apparent. However, the dose and type of chlorophyllides were not described in the study.

Several studies were peformed in *Bombyx mori* and demonstrated the anti-viral and anti-microbial activity of chlorophyllide *a*. Hayashita et al. demonstrated that the antiviral activity of chlorophyllide *a* is owed to its interaction with its interacting protein in *Bombyx mori* [160]. Chlorophyllide *a* was obtained from the acetone-soluble fraction of spinach leaves and hydrolyzed with chlorophyllase. The authors incubated chlorophyllide *a* and midgut protein precipitates from mulberry-raised silkworm larvae, *Bombyx mori*. The results suggest that chlorophyllide *a* could interact with a protein that exists in the digestive juice of the mulberry-raised silkworm larvae and that this interaction generates red fluorescent protein. The red fluorescent protein exhibits antiviral activity against the silkworm nuclear polyhedrosis virus. In addition, the antimicrobial activity of chlorophyllide *a* was reported by Pandian et al. [161]. Chlorophyllide *a* was prepared from fresh spinach using chlorophyllase. The author observed that chlorophyllide *a* binds with an epithelial cell membrane 252-kDa protein (P252) from the *Bombyx mori* midgut. When 50 μM of chlorophyllide *a* was used to form a complex with P252, the complex presented antibacterial activity against *Escherichia coli*, *Serratia marcescens*, *Bacillus thuringiensis*, and *Saccharomyces cerevisiae* with EC_50_ values of 2.82, 2.94, 5.92, and 21.6 μM, respectively. Furthermore, Chen et al. characterized a chlorophyllide *α*-binding protein that was highly and exclusively expressed in the midgut of the silkworm, *Bombyx mori* [162]. Chlorophyllide *α*-binding protein was specifically and highly expressed in the apical of midgut epithelial cells as a secretory protein. Sequence similarity was found between P252 [161] and chlorophyllide *α*-binding proteins. To determine the expression level of chlorophyllide *α*-binding protein and infection conditions, the authors further infected silkworms with silkworm nuclear polyhedrosis virus and *E. coli* [163]. Results showed that the expression levels of chlorophyllide *α*-binding protein were decreased in infected silkworms. This study demonstrated that chlorophyllide *α*-binding protein was supposed to bind the pathogens and then inhibit further infection. In addition, Manjunatha et al. purified red fluorescent protein from the midgut of Kolar Gold silkworms and confirmed their anti-microbial and anti-viral activity [163]. Red fluorescent protein was purified and collected from midgut juice under ultraviolet light. Antiviral and antimicrobial activities of purified red fluorescent protein against BmNPV, *Escherichia coli*, *Klebsiella pneumoniae*, *Bacillus subtilis*, and *Phytophthora meadii* were demonstrated [164]. Red fluorescent protein from the Kolar Gold silkworm showed homology with the chlorophyllide *α*-binding protein of *Bombyx mori*. Hu et al. further fed *Morus alba* into *Bombyx mori* for 26 days to examine the profiles of chlorophyllide in the midgut [164]. Chlorophyllide *a* was preferentially bound to the midgut of *Bombyx mori* [165]. Administration of 400 nmol/g chlorophyllide significantly inhibited larval growth of *S. litura*. Therefore, chlorophyllide *a* may be involved in defense systems of insect herbivores. It has been reported that chlorophyllides are phototoxic. Currently, the mechanism concerning detoxification of chlorophyllide in herbivores and the precise function of chlorophyllide α-binding protein remains unknown. We suspect that chlorophyllide may interact with chlorophyllide *α*-binding protein to form a complex (red fluorescent protein in *Bombyx mori* under light). This red fluorescent protein exerted antiviral and antimicrobial activities, indicating that chlorophyllide may be involved in detoxification or defense systems.

Hsu et al. studied the protective effects of chlorophyllide on hydrogen peroxide-induced DNA damage ex vivo in human lymphocytes [100]. Chlorophyllides *a* and *b* were prepared from chlorophyll in spinach and hydrolyzed by chlorophyllase from the *Ficus macrocarpa* leaf. Chlorophyllide *a*/*b* (5, 20, or 50 μM) was incubated with lymphocytes for 30 min and then exposed to 10 μM hydrogen peroxide. Cells were collected to assess the level of DNA damage. Results revealed that chlorophyllide *a* and *b* at 5–20 μM decreased hydrogen peroxide-induced DNA single-strand breaks and oxidized nucleosides. However, chlorophyllide *a*/*b* did not present protection against 50 μM hydrogen peroxide-induced DNA damage. Their antioxidative activity was assessed using a 1,1-diphenyl-2-picrylhydrazyl radical scavenging assay. The authors observed that chlorophyllide *a*/*b* did not show scavenging ability with an IC_50_ value greater than 800 μM. Hsu et al. further clarified the inhibitory effects of chlorophyllides on aflatoxin B1-induced DNA adducts in murine hepatoma (Hepa-1) cells [99]. Chlorophyllide *a*/*b* was extracted from spinach and hydrolyzed by chlorophyllase isolated from the leaf of *Ficus macrocarpa*. Pre-treatment with chlorophyllide *a*/*b* at 0, 5, 20, or 50 μM in Hepa-1 cells for 24 h was then exposed to aflatoxin B1 (5 and 10 ng/mL). Aflatoxin B1-DNA adduct formation was examined by enzyme-linked immunosorbent assay. They observed that chlorophyllides inhibited 5 or 10 ng/mL of aflatoxin B1-DNA adduct formation in a dose-dependent manner. 50 μM of chlorophyllides showed significant inhibition. To demonstrate that chlorophyllide *a*/*b* could directly bind to aflatoxin B1-DNA adduct, a washout experiment was performed. Cells were pre-treated with chlorophyllide *a*/*b* for 24 h and then exposed to aflatoxin B1. The authors reported that chlorophyllide *a*/*b* did not inhibit the formation of the aflatoxin B1-DNA adduct in the wash-out experiment, indicating that chlorophyllides (50 μM) directly trapped aflatoxin B1 and eliminated the aflatoxin B1-induced effect.

Lamontagne et al. identified the chlorophyllide obtained from a compound library that presented antiviral activity [166]. This compound library contained 2000 food and drug administration (FDA)-approved compounds or natural products. In brief, cultured cells were treated with 10 μM chlorophyllide for 6 days, and the secreted hepatitis B virus DNA was detected in HepG2.215 and viral particles were detected in HepDE19 cells. They observed that chlorophyllide significantly decreased the hepatitis B virus DNA with an IC_50_ of 1.5 μM without affecting the cell viability. Treatment with chlorophyllide for 5 days decreased viral particles in a dose-dependent manner in HepDE19 cells. Guo et al. determined the antiviral effects of chlorophyllide and chlorin e6 [167]. The chlorophyllide used in this study is a chlorophyllide Cu complex and Na salt, and chlorin e6 is a metal-free chlorophyllide-like molecule. Chlorophyllide or chlorin e6 were incubated with HepDE19 cells for 8 days, and hepatitis B virus DNA and virion particles were then determined. They observed that chlorophyllide or chlorin e6 has an inhibitory effect on virion-associated hepatitis B virus DNA. Chlorophyllide (3–10 μM) decreased the level of intact enveloped virion particles in a dose-dependent manner, but did not affect viral DNA. The EC_50_ of chlorin e6 in human immunodeficiency virus, dengue virus, marburg virus, junin viruses, and herpes simplex virus type 1 was approximately 2–5 μM, while that against the herpes simplex virus and dengue virus was 20.93 μM and 0.3 nM, respectively.

Chlorophyllide was included in a US Drug Collection library that contained 1040 FDA-approved compounds. Two studies observed that chlorophyllide has different effects on cell growth. Ou et al. used a zebrafish model to screen compounds that have ototoxic effects against hair cells [168]. The authors showed that chlorophyllide has ototoxicity that causes hair cell death in zebrafish lateral line. Musdal et al. examined inhibitors of glutathione transferase P1-1, an enzyme that was overexpressed in cancer cells and correlated to drug resistance [169]. The results showed that the enzyme activity of glutathione transferase P1-1 was inhibited in the presence of chlorophyllide (IC_50_: 2.3 μM). However, the author cataloged chlorophyllide as a food-coloring additive and did not perform further study.

It has been discovered that chlorophyllide may have antipathogenic activities in plants through the accumulation of reactive oxygen species. Kariola et al. observed that chlorophyllide was involved in the defense against *Alternaria brassicicola* [170]. The authors targeted Arabidopsis chlorophyllase with RNA interference to silence the expression of the chlorophyllase gene. They discovered that the silencing of chlorophyllase caused the accumulation of reactive oxygen species and increased the resistance to *Erwinia carotovora* (bacterial necrotroph), but enhanced the susceptibility to *Alternaria brassicicola* (necrotrophic fungus). Hu et al. further examined the hypothesis that chlorophyllide and chlorophyllase formed a defense system against herbivores [164]. Chlorophyllide *a* was extracted and purified from the leaves of *Glebionis coronaria*. Chlorophyllase-overexpressing plants or purified chlorophyllide (200 or 400 nmol/g fresh weight) were fed to *Spodoptera litura* larvae, and the survival rate was observed to determine the toxicity of chlorophyllide. The results indicated that purified chlorophyllide slightly decreased the survival rate of larvae. Normally, plant cells could scavenge the excess of reactive oxygen species [171,172,173].

Souid et al. discovered that 13^1^-oxophorbine protopheophorbide *a*, protopheophorbide *a*, and chlorophyllide *a* from *Ziziphus lotus* have antiproliferative activity against the human breast cancer cells MDA-MB-231 and MCF-7 [174]. Chromatography was used to extract and isolate these compounds: 13^1^-oxophorbine pheophorbide *a*, protopheophorbide *a*, and chlorophyllide *a* from *Ziziphus lotus* leaves. The viability of MDA-MB-231 cells was determined after treatment with chlorophyllide for 72 h. The authors observed that chlorophyllide *a* inhibited the viability of MDA-MB-231 cells with IC_50_ values of 14.5 µM; however, chlorophyllide *a* did not show any inhibitory effects on MCF-7 cells. Based on a wound healing assay, they reported that 5 µM of chlorophyllide *a* present 27% of inhibition on MDA-MB-231 cells, while that of protopheophorbide *a* was 74%.

The cytotoxicity of chlorophyllides has been reported by our group [175]. Crude extracts from plant leaves were treated with CrCLH1 chlorophyllase to prepare chlorophyllides. Chlorophyllides were then used to determine inhibitory effects against breast cancer cells (MCF-7 and MDA-MB-231), hepatocellular carcinoma cells (Hep G2), colorectal adenocarcinoma cells (Caco2), and glioblastoma cells (U-118 MG) by MTT (3-(4,5-dimethylthiazol-2-yl)-2,5-diphenyl tetrazolium bromide). We observed that chlorophyllides in crude extracts from sweet potato were the most effective against all cancer cell lines tested. The contents of chlorophyllides in crude extracts were approximate 10% (the concentrations of chlorophyllide *a* and *b* were ~7.7% and ~2.3%, respectively). The IC_50_ values of chlorophyllides in MDA-MB-231 and MCF-7 cells were 82.9 and 122.29 μg/mL, respectively. The results indicated that the contents of chlorophyllides from sweet potato were closely correlated to their cytotoxic activity. In addition, we also observed that the cytotoxic effects of chlorophyllides was better than those of chlorophylls or commercial chlorophyllin. Therefore, crude chlorophyllides from sweet potato may have cytotoxic effects against cancer cell lines. We further designed a facile method for chlorophyll purification by twice solvent extraction [176]. The purity of chlorophylls was increased by twice extraction and then used to obtain purified chlorophyllides. Our results showed that the purified chlorophyllides were active and may have a combination of effects with doxorubicin both in MCF-7 and MDA-MB-231 cell lines.

### 4.2. Applications in Photoactivity

The photosensitivity of chlorophyllides was determined by Tapper et al., who investigated the components in lucerne (*Medicago sativa*) leaf protein concentrates or purified pigments that led to photosensitivity [177]. Rats were fed a diet containing 5% and 20% leaf protein concentrates or purified pigments and illuminated at 30 Wm^−2^ for 5–6 h for 12 days; skin lesions were used as indicators of photosensitivity. Lucerne leaf protein concentrates or purified pigments were subjected to heat at 70 °C for 55 min, or 90 °C/cool down within 2 min. The combined levels of pheophorbide *a* and chlorophyllide *a* from leaf protein concentrates treated at 70 °C and 90 °C were 6.3 and 1.9 mg/g dry weight, respectively. The results indicated that leaf protein concentrates treated at 90 °C showed no deaths or skin lesions in rats. Rats with 20% leaf protein concentrates that were treated at 70 °C died before the development of skin lesions. 4 mg/g dry weight of purified chlorophyllides or 0.5–2 mg/g dry weight of pheophorbide *a* were observed in the skin lesions of the surviving rats. Therefore, chlorophyllides or pheophorbide *a* were strongly photosensitizing pigments. Gerola et al. investigated the antimicrobial effects of an analog Zn-chlorophyllide *a* [178]. Zn-chlorophyllide *a* was prepared as follows: chlorophyll *a* was extracted from spinach and replaced with Zn in a mixture of glacial acetic acid and chloroform, and then subjected to acid hydrolysis to obtain Zn-chlorophyllide *a*. The authors observed that the photostability of Zn-chlorophyllide *a* was higher than that of chlorophyll *a*. The antimicrobial effect of Zn-chlorophyllide *a* (20, 40, 60, and 100 μg/mL) against *Staphylococcus aureus*, *Escherichia coli*, *Candida albicans*, and *Artemia salina* was investigated. The suppression ratio (in the dark) of Zn-chlorophyllide *a* (20–100 μg/mL) against *Staphylococcus aureus* was 79.6–99.7%. Zn-chlorophyllide *a* led to the death of *Escherichia coli* in the dark at all concentrations tested. The killing effect of Zn-chlorophyllide *a* on *Candida albicans* resulted in approximately 40% of deaths in the dark. The cytotoxicity of Zn-chlorophyllides (1, 5, 10, 50 μg/mL) against *Artemia salina* was 38–100%. These results showed that Zn-chlorophyllide *a* could be an excellent bactericide in the dark. The same authors formulated Zn-chlorophyllide *a* with polymeric micelles of F-127 pluronics and liposomes of zwitterionic phospholipid dipalmitoylphosphatidylcholine [179]. Zn-chlorophyllide (2 μmol/L) was used as a monomer and loaded into F-127 micelles or liposomes. The complexes were then used to evaluate the photodynamic inactivation of *S. aureus*. The results indicated that the death rate of Zn-chlorophyllide in F-127 decreased by 75% compared to that in the dark. The death rate of Zn-chlorophyllide in liposomes in the light decreased by 25% compared to that in the dark. In conclusion, the photoinactivation of Zn-chlorophyllide against *Staphylococcus aureus* in liposomes was higher than that in micelles.

Several applications of chlorophyllides in engineering have been reported. For example, in 2005, Tadini-Buoninsegni et al. designed an alkanethiol-coated mercury electrode, and chlorophyllide was used as the film adsorbed on the electrode [180]. The source of chlorophyllide was not mentioned in this study. The chlorophyllide film was assembled directly on mercury from hexane. The authors observed that when chlorophyllide was illuminated with a red light in an aqueous solution (0.1 M KCl, pH 8.5), the electrochemical reaction of chlorophyllide generated a photocurrent. At negative potentials, the chlorophyllide film mediated electron transfer from the electrode to the water. They also reported that chlorophyllide on the electrode undergoes electroreduction, which generates a reorganization energy after being photoexcited.

Hamer et al. reported a new method for the synthesis of gold nanoparticles, modified with polyallylamine-zinc-chlorophyllide *b* composite, and used the composite as a sensor [181]. Chlorophyllide *b* was obtained from spinach using methanol extraction. Polyallylamine, a cationic polyelectrolyte combined with chlorophyllide *b*, forms an adsorbed film of charged polymers. Ultraviolet–visible (UV–Vis) spectroscopy of polyallylamine-chlorophyllide *b* showed an intense band at 660 nm, suggesting that chlorophyllide *b* at the center of the polymer was optically reactive. UV–Vis spectra of polyallylamine-zinc-chlorophyllide *b* increased in the presence of Zn. Polyallylamine-chlorophyllide *b* or polyallylamine-zinc-chlorophyllide *b* were then used as materials to prepare gold- or silver-polyallylamine-chlorophyllide *b* composite nanoparticles. The authors observed that gold nanoparticles interacted with polyallylamine-chlorophyllide *b* and then increased the extinction coefficient. In addition, gold nanoparticles stabilized by polyallylamine, owing to interparticle distance, were maintained. Therefore, chlorophyllide acting as an optical center may be applied in biosensors. In fact, the chlorophyllide *b* mentioned in this research was a demetalized form, and we suggested that chlorophyllide *b* and chlorophyll *b* should be modified as pheophorbide *b* or pheophytin *b*, respectively. Hamer et al. further studied polyallylamine-chlorophyllide *b* as a sensor to detect carbon dioxide [182]. Polyallylamine-chlorophyllide *b* forms a green polymer with two absorption bands at 402 and 660 nm. The green polymer combined with CO_2_ to form a hydrogel excited at 545–580 nm and 460–490 nm. In addition, UV–Vis spectra of polyallylamine-chlorophyllide *b*-CO_2_ presented a growing peak at 250 nm, which is a characteristic of the carbamate group. Results demonstrated that polyallylamine-chlorophyllide *b*-CO_2_ presented a stable fluorescent response in a CO_2_ dose-dependent manner and allowed the detection of 2–5 part per million (ppm) of CO_2_.

Chlorophyllide, chlorophyll, and sodium copper chlorophyllin have been used extensively as colorants in food, medicine, and pharmaceutical applications. For example, chlorophyllide, which has a function in pigment concentration and film thickness, was added to artificially colored edible films [183]. Ten percent of chlorophyllide, sorbitol, and gelatin were cast into a fil-forming solution to produce gelatin films. The addition of chlorophyllide to the film did not affect the mechanical resistance, water-solubility, or translucence, but it increased the gloss on the film surface and decreased surface roughness. Chlorophyllide significantly increased the color of the films without affecting physical properties in a dose-dependent manner. Therefore, chlorophyllides might be considered as an ingredient in edible film.

Tostado-Plascencia et al. synthesized carbon nanotubes functionalized with chlorophyllide derivatives and characterized their optical properties [184]. *Hibiscus tiliaceus* leaves were mixed with acetone and petroleum ether to extract chlorophyll *a*. Chlorophyll *a* solution was then incubated with potassium hydroxide to synthesize chlorophyllide *a*, which was used to synthesize carbon nanotubes. The results indicated that covalent bonds were formed between chlorophyllide and carbon nanotubes, absorbance peaks were shifted, and signals from UV–Vis spectroscopy, Raman spectroscopy, and fluorescence spectroscopy were quenched. The carbon nanotube-functionalized chlorophyllide *a* showed a compact and flat surface with cluster and agglomerated morphologies. Therefore, chlorophyllide-functionalized carbon nanotubes could be applied in gas sensing or solar energy.

Obtaining a large amount and purified chlorophyllides is necessary for functional studies. We propose future perspectives:

1. New recombinant enzymes with high catalytic activity should be developed.
2. Isolation and purification of chlorophyllides should be optimized.
3. New preservation technology for chlorophyllides should be developed.
4. The detailed mechanism of chlorophyllides interacting with other proteins or compounds should be investigated, which might help us to understand the activity of chlorophyllides (e.g., cytotoxicity or antiviral activity).

## 5. Conclusions

Chlorophyllides and its derivatives have attracted the attention of a wide spectrum of researchers since the 19th century. Applications of chlorophyllides and their derivatives are emerging topics in medicines, the food industry, and science. Generally, chlorophyllides play a fundamental and important role in plants. This review summarizes and integrates the preparation, purification, and applications of chlorophyllides. Recently, cutting-edge instruments, such as cryogenic electron microscopy, have caught our attention, assisting in analyzing the molecular docking process of chlorophyllide. The understanding of chlorophyllide-affected protein structures or cellular ultrastructure might provide solutions for serious human disease problems. For a comprehensive understanding of chlorophyllides, the crystallographic structure of the complex between chlorophyllides and other proteins, viruses, and cancer cells should be elucidated. In addition, bioactivity-oriented screening would have benefits in revealing new applications of chlorophyllides. We suggest that the detailed mechanism of chlorophyllides in medical therapy, disease prevention, or health maintenance should be further studied, and would be useful in making chlorophyllides beneficial for human welfare.

## Figures and Tables

**Table 1:** The molecular formula, molecular weight, and structure of chlorophyllides.

**Name**	**Chlorophyllide *a***	**Divinyl Chlorophyllide *a***	**Chlorophyllide *b***	**Chlorophyll(ide) *c*_1_**	**Chlorophyll(ide) *c*_2_**
**Molecular formula**	C_35_H_34_MgN_4_O_5_	C_35_H_32_MgN_4_O_5_	C_35_H_32_MgN_4_O_6_	C_35_H_30_MgN_4_O_5_	C_35_H_28_MgN_4_O_5_
**Molecular weight (g/mol)**	615	613	629	611	609
**Structure**	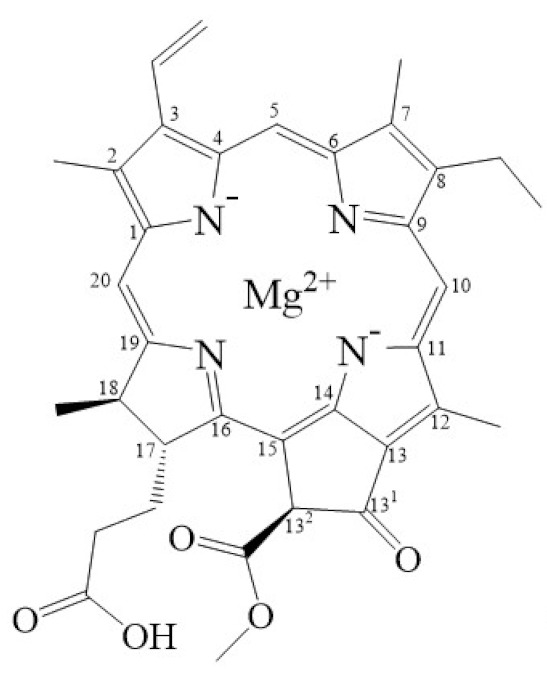	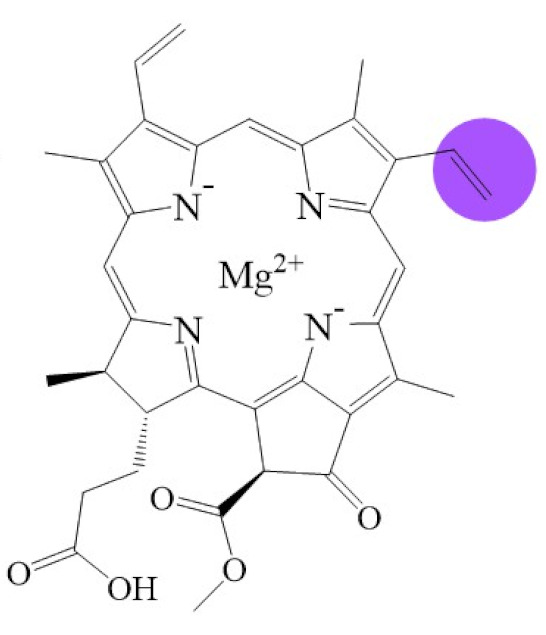	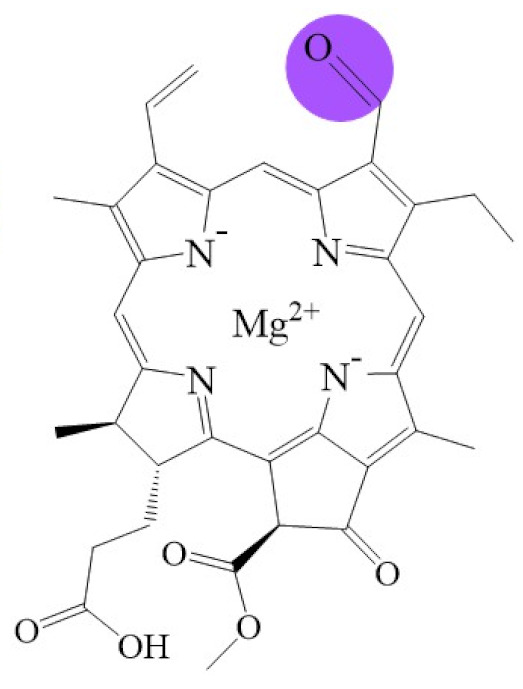	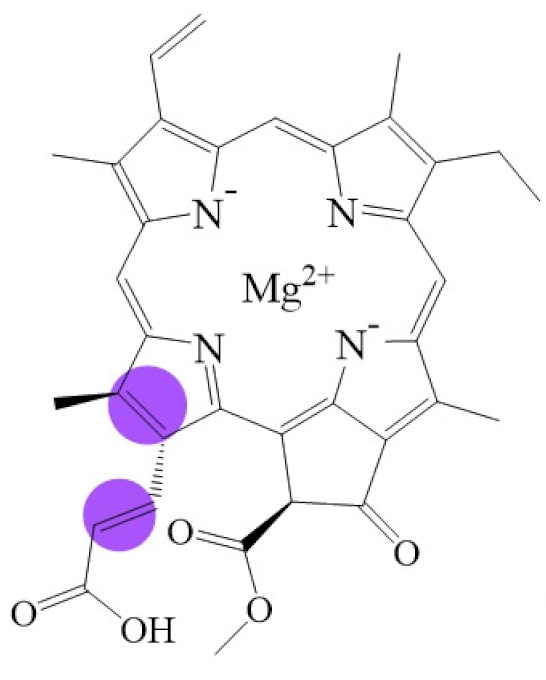	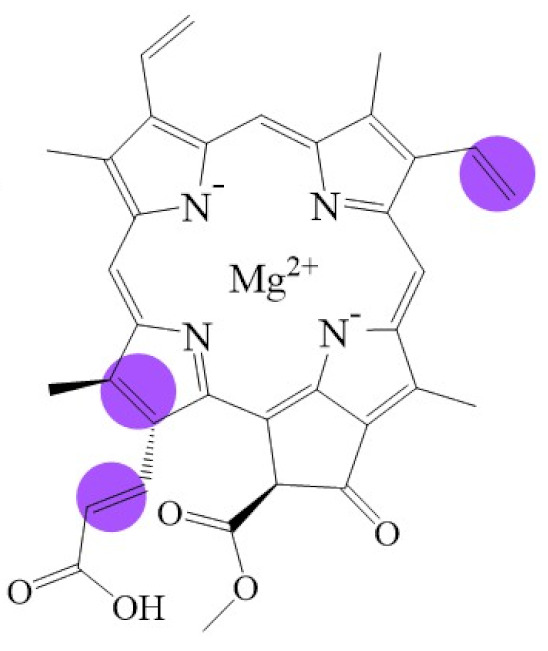
**Name**	Chlorophyll *c*_3_	8-ethyl chlorophyll *c*_3_	Chlorophyll *c*_CS-170_	Chlorophyllide *d*	Chlorophyllide *f*
**Molecular formula**	C_36_H_31_MgN_4_O_5_	C_36_H_34_MgN_4_O_5_	C_36_H_32_MgN_4_O_5_	C_34_H_32_MgN_4_O_6_	C_35_H_32_MgN_4_O_6_
**Molecular weight (g/mol)**	653	655	655	617	629
**Structure**	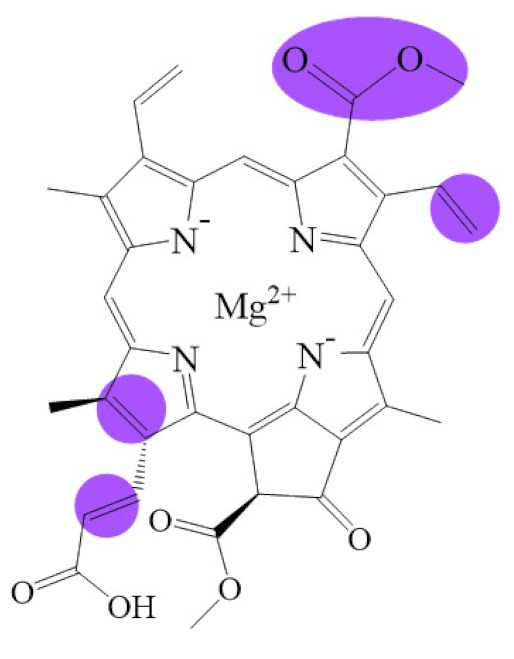	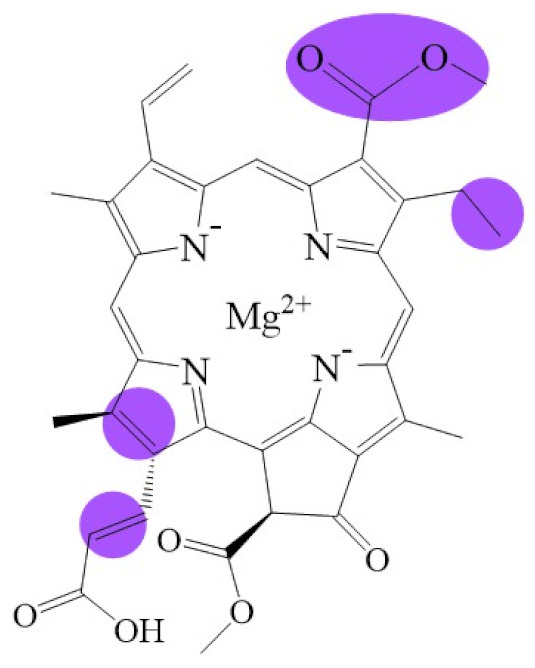	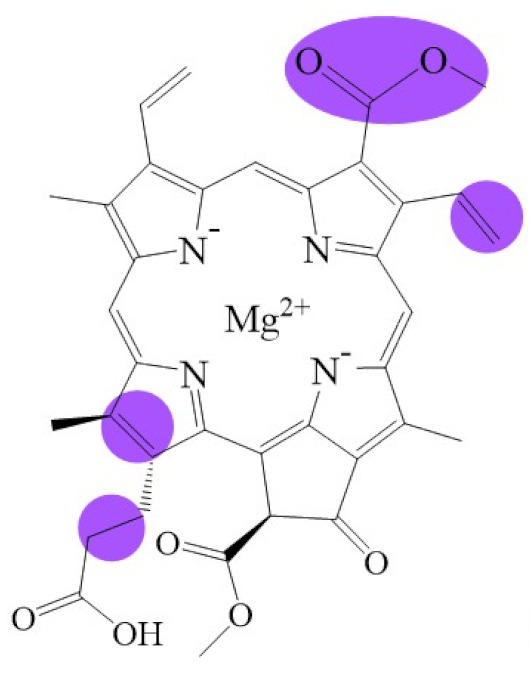	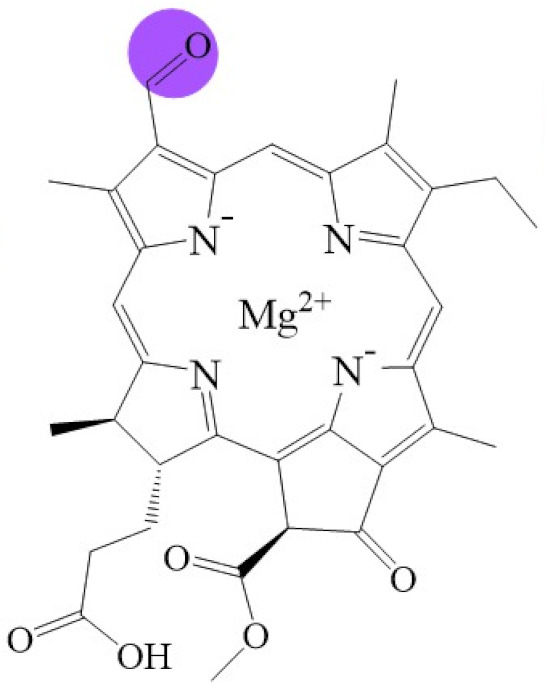	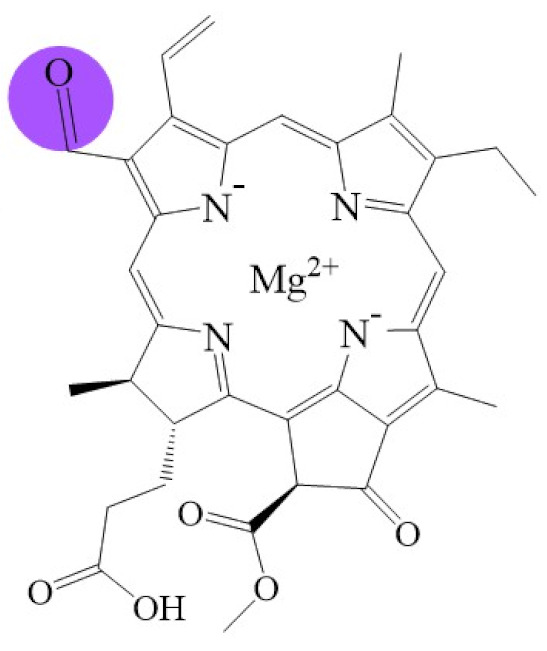

Note: The structure differences of chlorophyllide derivatives to chlorophyllide *a* are highlighted with spots. The IUPAC numbering system was used.

**Table 2:** Some examples of chlorophyllides synthesis from protochlorophyllide and chlorophyll.

Starting Materials	Enzyme	Plant Source	Affected Factor	Yield	References
Protochlorophyllide *a*	NADPH: protochlorophyllide oxidoreductase	Etiolated leaves	Light (+)	N/A	[73]
		Wheat leaves	*δ*-aminolevulinic acid (5 mM)/light (+)	2.5–18 μg/g fresh weight at 1–57 μW/cm^−2^ of light for 15 min	[75]
		Wheat (*Triticum aestivum* L., cv. Kosack) seed	Norflurazon, inhibitor of carotenoid biosynthesis (+)	Protochlorophyllide: 39 μg/g at norflurazon-treated leaves (100 μg/L), the ratio of chlide/protochlorophyllide was increased	[93]
		Barley leaves (*Hordeum vulgare*)	Water (+) on Chlorophyll(ide)_684_ (−) on Chlorophyll(ide)_676_	Chlorophyll(ide)_676_ was increased 28.4–92.5% in 40% and 78% desiccated leaves	[97]
		Barley seeds (*Hordeum vulgare* L. Zaoshu No. 3)	Nitric oxide (−)	Nitric oxide inhibited chlorophylls synthesis (49–58% decline in barley and 21–24% decline in Arabidopsis)	[98]
Chlorophyll	Chlorophyllase	*Phaeodactylum tricornutum*	50% acetone at 20 °C (+)	100%	[49]
		*Heruckum* leaves	Heat treatment between 60–75 °C (+)	10–70%	[50]
			80% acetone at 1 °C (+)	100%	
		*Ficus macrocarpa* leaves	80% acetone (+)	95%	[99,100]
		French bean leaves	*δ*-aminolevulinic acid (+)	N/A	[101]
	*Citrus sinensis* chlorophyllase	*Nicotiana tabacum*		1.8 fold increased	[102]
	*Chlamydomonas reinhardtii* chlorophyllase			Catalytic efficiency: 9.89 (s^−1^ μM^−1^)	[57]
	*Brassica oleracea* chlorophyllase			Catalytic efficiency: 15.51 × 10^−5^ (s^−1^ μM^−1^)	[56]
	Cyanobacterium *Cyanothece* sp. ATCC 51142 chlorophyllase			Specific activity of enzyme: 75.60 (U/mg)	[58]
	*Oscillatoria acuminata* PCC 6304 chlorophyllase			Catalytic efficiency: 15.51 × 10^−5^ (s^−1^ µM^− 1^)	[103]

N/A: not available; chlorophyll(ide)_676_ is characterized by fluorescence absorption and emission maxima at 668 nm and 676 nm, respectively; chlorophyll(ide)_684_ is characterized by fluorescence absorption and emission maxima at 674 nm and 684 nm, respectively; chlorophyll(ide)_676_ and chlorophyll(ide)_684_ are partially esterified chlorophyllide species.

**Table 3:** Some examples of chlorophyllides purification by solvents.

Purification by Solvents	Sources	Yield	References
Membrane suspension was added to a mixture containing acetone (or methanol) and 0.1 M ammonium hydroxide. After being mixed and centrifuged, these combined supernatants was added to petroleum ether or *n*-hexane.	Plasma membranes and thylakoid membranes of *Anucystis nidulans*	N/A	[133]
Extracted diethyl ether/ethanol (1:1) and repeatedly washed with 20% ethanol (10 mM Tricine–NaOH, pH 8).	*R. capsulatus* CB1200 cultured in Tween 80 supplemented growth medium	Chlorophyllide *a*: 7 mg/L	[134]
Barley leaves were extracted in 80% acetone (acetone/50 mM Tricine–NaOH, pH 8, 80:20, *v*/*v*), and the suspension was repeatedly extracted with *n*-hexane and diethyl ether/ethanol (1:1). The suspension was then repeatedly washed with 20% ethanol (10 mM Tricine–NaOH, pH 8).	Barley leaves (*H. vulgare* L.)	Chlorophyllide *a*: 17 μg/g fresh weightChlorophyllide *b*: 5 μg/g fresh weight	

N/A: not available.

**Table 4:** Some examples of chlorophyllides purification by chromatography.

Chromatography	Elution	References
Column chromatography with sugar or cellulose powder packaged column	N/A	[48]
Column chromatography with DEAE-Sepharose CL-6B column	Elution: acetone/H_2_O (4:1, *v*/*v*), 1% ammonium acetate	[135]
High-performance liquid chromatography (HPLC), silicic acid column coated with dodecyl residues	Methanol: tetrabutylammonium phosphate: methyl ethyl ketone (70:30:6; *v*/*v*/*v*) Elution: 70% methyl alcohol with methyl ethyl ketone	[136]
Reverse-phase HPLC system with column packed with octadecyl-silane bonded 5-μm ODS-Hypersil	Mobile phases contain 1.5% tetrabutylammonium acetate and 7.7% ammonium acetate in water:water:methanol (10:10:80; *v*/*v*/*v*); acetone:methanol (20:80; *v*/*v*) in the gradient elution	[137]
HPLC with a Shimadzu LC-3A chromatograph using a polyethylene column	Acetone in water: 50% (*v*/*v*)	[138]
HPLC with reversed-phase Spherisorb ODS-2 column	The mobile phases contained 80% methanol in ammonium acetate solution and 80% methanol in acetone	[139]
HPLC with reversed-phase C18 column and photodiode-array detector	The mobile phase contained acetonitrile-methanol (70:30, *v*/*v*) and increasing proportions of dichloromethane	[140]
HPLC with a monolithic C18-bonded silica rod column	The mobile phases contained 80% methanol in 0.025 M aqueous pyridine solution (pH 5 with acetic acid) and 80% methanol in acetone	[142]
HPLC−Diode Array Detection−Mass Spectrometry with an HyPURITY C18 column	The solvent system was consisted of water, methanol, acetonitrile and acetone with a gradient condition at 1 mL/min of flow rate	[144]
A high-throughput method that combined with HPLC and time-of-flight mass spectrometry with a C18 Spherisorb ODS-2 column	The mobile phases: water/ion pair reagent/methanol (1:1:8, *v*/*v*/*v*) and methanol/acetone (1:1, *v*/*v*), 1 M ammonium acetate in water was used as the ion reagent	[145]

## Abbreviations

bp	base pairs
CAO	chlorophyllide *a* oxygenase
chlorophyllase	chlorophyll chlorophyllidohydrolase
DVR	3,8-divinyl protochlorophyllide *a* 8-vinyl reductase
DPOR	dark-operative protochlorophyllide oxidoreductase
FDA	food and drug administration
FNR	ferredoxin–NADP^+^ oxidoreductase
GA3	gibberellin-A3
HPLC	high-performance liquid chromatography
kDa	kilodalton
LC	liquid chromatography
LPOR	light-operative protochlorophyllide oxidoreductase
protochlorophyllide *a*	monovinyl protochlorophyllide *a*
nm	nanometer
NADPH	nicotinamide adenine dinucleotide phosphate
ppm	part per million
UV–Vis	ultraviolet–visible
